# Factors influencing healthy role models in medical school to conduct healthy behavior: a qualitative study

**DOI:** 10.5116/ijme.5ff9.9a88

**Published:** 2021-01-22

**Authors:** Michael A. Leman, Mora Claramita, Gandes R. Rahayu

**Affiliations:** 1 School of Dentistry, Faculty of Medicine, Universitas Sam Ratulangi, Manado, Indonesia; 2 Department of Medical, Health Professions Education and Bioethics, Faculty of Medicine, Public Health, and Nursing, Universitas Gadjah Mada, Yogyakarta, Indonesia

**Keywords:** Healthy role model, healthy behavior, medical teacher, medical school

## Abstract

**Objectives:**

This study aimed to identify the factors that support or inhibit medical teachers as healthy role models in medical school to conduct healthy behavior.

**Methods:**

This qualitative study involved semi-structured in-depth interviews with medical teachers categorized as healthy role models in a medical school from a previous survey. Ten medical teachers were selected using purposive sampling. Three medical teachers were interviewed by direct meetings, and the remaining were phone interviewed, with one interview facilitated by chat using WhatsApp. Transcribed interviews were coded openly. Themes were finalized through discussion and debate to reach a consensus.

**Results:**

Two themes were identified: perceived facilitators and perceived barriers, which were classified into four categories and 13 subcategories: intrinsic facilitators (motivation, conscious awareness, having physical limitations, knowledge, and economic reasons); extrinsic facilitators (the impact on doing a particular job, feedback, time, and environment); intrinsic barriers (the lack of self-motivation and having physical limitations); and extrinsic barriers (the burden of responsibilities for being medical teachers and environment).

**Conclusions:**

Factors that support and inhibit medical teachers as healthy role models in medical school are influenced by intrinsic and extrinsic factors. This result could be used by medical schools to design appropriate interventions to help medical teachers as healthy role models in conducting healthy behavior. More studies are needed to explore other factors that influence medical teachers to conduct healthy behavior. During the COVID-19 pandemic, healthy role models in medical schools are vitally important and significantly contribute to the overall health of a nation.

## Introduction

Medical school, as a part of the university, is a context for implementing the Health-Promoting University (HPU) initiative. First implemented at The University of Central Lancashire in 1995, this initiative aims to create a school environment and culture that integrate health values in their teaching and learning activities as well as in educational policies. Providing healthy role models for students by both faculty and staff is the main characteristic of any school that has successfully implemented HPU.^1^

The existence of a school environment and culture that integrates health values helps to develop the characteristics of medical teachers, staff, and students as ‘agents of change’ for a healthy lifestyle in the community. Medical teachers and students who are from a school that has implemented HPU should be able to act as healthy role models by conducting healthy behavior not only among themselves but also in the surrounding community.^1^^-^^3^ To make this happen ideally, practicing healthy behavior in their daily life is one of the main ways to fulfill their role as a healthy role model in the community. Dooris^1^ explained that this personal health empowerment is one of six main focuses of HPU implementation in the school.

The term healthy role model in medical school has not been found in publications. Therefore, to explore the definitions and characteristics of a healthy role model in medical school, we previously conducted a grounded theory study.^4^ We interviewed and communicated with 48 medical teachers from various backgrounds of ‘health professions education’, ‘health education and behavior’/ ‘health education and promoter,’ ‘general practitioners’/’family medicine’, ‘adolescent health’, ‘internal medicine’, and ‘cardiology-vascular medicine’. We also conducted three focus group discussions with medical students. We found that a healthy role-model in a medical school is a person who is seen as: physically, socially, mentally, and spiritually healthy; internalized healthy behaviors in their life; willing to promote healthy lifestyles; and are life-long learners. Practicing healthy behavior is one of the descriptions of the first and second characteristics of a healthy role model in a medical school, according to our previous study.^4^

Because a medical school is where the future physicians are produced, the primary concerns of medical schools should be producing not only a competent physician but also a healthy physician who can model healthy behaviors to their patients and the surrounding community.^5^ The medical school should provide a safe place to practice healthy behaviors and develop these healthy characteristics among staff and students.^1^ Our previous grounded theory^4^ study also found that medical teachers are the first and primary person expected to be healthy role models in medical school. The previous participants said that the medical teachers are the sources of healthy role models for both the students and academic staff. Furthermore, McAleer and Roff^6^ described how medical teachers are the main people who students will interact with when they first arrive at medical school. Accordingly, the healthy characteristics that their medical teachers have will influence the students’ future characteristics as physicians.

In medical school, most students' learning occurs formally in the classroom, but many students also learn by examples through modeling from their teachers.^7^ Learning through modeling is a learning method that is explained by the social cognitive theory.^8^ Ficklin and colleagues^9^ stated that modeling is an effective learning method in medical schools. In modeling, medical students must observe what is modeled, create a mental representation of it, reproduce what was modeled, monitor what they perform, and motivate themselves to model the behavior continuously.^8^ It is well-established that learning how to behave in a healthy manner can also be effectively done through modeling.

However, several studies^10^^,^^11^ in medical schools showed that many medical teachers are not aware of their role as role models in medical school. They rarely realized that they are a model of healthy behaviors for their students. Therefore, we previously surveyed the characteristics of medical teachers as healthy role models in one of the medical schools in Indonesia.^12^ Before conducting the previous survey, we developed a questionnaire to measure the characteristics of a healthy role model maintained by medical teachers. The categories and subcategories that we obtained from grounded theory were used as a pooled item inventory to develop this questionnaire. We classified medical teachers as a healthy role model in medical school into two groups. The medical teachers who have all characteristics of healthy role models are defined as active healthy role models. The medical teacher who has most of the characteristics of a healthy role model in medical school but did not perform healthy modeling adequately or actively is defined as passive healthy role models. We asked our participants to make a self-assessment of what characteristics they have as a healthy role model in medical school. We found that 60 of 79 participants who participated in the survey have not modeled healthy behavior effectively; thus, we classified them as passive healthy role models in medical school. To explore the reasons behind what we found in the previous survey, we next conducted this qualitative study to identify the factors that could support and inhibit the medical teachers as a healthy role model in medical school in conducting healthy behaviors.

## Methods

### Study design, participants, and setting

This research used a qualitative design, since this study focuses on the experiences and perceptions of medical teachers, highlighting what factors support and inhibit them to perform healthy behaviors as a healthy role model in medical school.

This study was conducted at the Faculty of Medicine, Public Health, and Nursing, Universitas Gadjah Mada, in Yogyakarta with medical teachers who had participated in our previous survey.^12^ From the previous survey, there were 79 medical teachers, including 19 medical teachers categorized as active healthy role models and 60 passive healthy role models. We selected the participants by purposive sampling in each group using a randomizer research software from the Social Psychology Network.^13^

An invitation to participate in this study was sent out to the selected participants. The first author (MAL) then contacted the participants by WhatsApp. The participants who did not respond in 14 days since the first day the invitation was sent were excluded. Ten medical teachers agreed to participate, including five active healthy role models, and five passive healthy role models. All of these medical teachers have a different field background in medicine. They include staff from the Department of Anesthesiology and Intensive Therapy, Department of Ear, Nose, and Throat (ENT), Department of Forensic Medicine and Medicolegal, Department of Pharmacology and Therapy, Department of Health Nutrition, Department of Neurology, Department of Microbiology, Department of Histology, Department of Health Policy and Management, and Department of Psychiatry.

Ethical approval was issued by the Medical and Health Research Ethics Committee Faculty of Medicine, Public Health and Nursing, Universitas Gadjah Mada, Indonesia, under their file number 0946 and 1217. All participants individually signed the informed consent forms regarding their agreement to participate according to the guidelines of brief descriptions to study subjects from the Medical and Health Research Ethics Committee Faculty of Medicine, Public Health and Nursing, Universitas Gadjah Mada.

### Data collection

Data collection was conducted from February to April 2020. We conducted semi-structured in-depth interviews to stimulate the participants to talk freely about their experiences as a healthy role model in conducting healthy behaviors. All participants received an explanation of the semi-structured in-depth interview process and gave their consent to participate. The semi-structured in-depth interviews were conducted using the guiding questions, as shown in Appendix 1. The list of questions was based on what was known from the literature concerning factors related to healthy behavior. The list was validated by three experts: one expert in health education and behaviors and two from a medical education background. All interviews were audio-recorded using a digital recorder after all participants completed the written informed consent forms.

The first three interviews were conducted in a quiet discussion room of the Faculty of Medicine, Public Health and Nursing, Universitas Gadjah Mada, where the participants work. Since all medical schools in Indonesia were ‘locked down’ in March 2020 due to the COVID-19 pandemic, the remaining interviews were conducted from home using telephone communication, except for one participant who preferred not to communicate by phone. This participant was facilitated by a chat using WhatsApp. We used the same list of questions from the interview guide to facilitate the communication with this participant. The chat communication was recorded and kept as screenshots by the interviewers.

The first author (MAL) conducted all interviews. MAL is a health care professional and had experience as the primary interviewer. MAL also did not work as a medical teacher in the Faculty of Medicine, Public Health and Nursing, Universitas Gadjah Mada, where the study took place. Therefore, MAL did not have any previous connection with the participants.

All interviews took 30-60 minutes and were transcribed verbatim by a transcriber service agent. MAL examined all transcripts by checking line by line to see the congruence between the transcripts and recordings. This process was supervised by the two other authors (MC and GRR). MC and GRR are health care professionals and have experience in conducting qualitative research. All audio records were listened to several times. MAL analyzed the transcripts repeatedly and rigorously under the supervision of MC and GRR. Data saturation was achieved when there were no new comments from the participants and agreed on by MAL, MC, and GRR.

### Data analysis

Transcripts were read and open coded by two coders: MAL, with the help of one independent coder (NW). NW is a health care professional with experience in conducting qualitative data analysis. The transcriptions were independently coded line by line by these two coders. Differences in codification were debated and solved through several discussions by the two coders. Through the discussion and debate, the identified subcategories, categories, and their associations that were clustered into themes were explored. In the process, the representative quotations were also discussed and selected. MC and GRR helped to clarify the results of codification made by the two coders. Differences in the codification were then debated by MC and GRR and solved through discussion until a consensus was reached. The subcategories, main categories, and the representative quotations were also reviewed and revised by MC and GRR. No new categories and subcategories emerged after the seven semi-structured in-depth interview transcripts were analyzed. The remaining transcripts were checked and used to ensure data saturation.

Credibility was achieved by conducting member checking. Memos and documents from the interviews and coding steps, which were kept by the first author, were also used to increase credibility. The subcategories and categories were derived from an inductive qualitative content analysis of participants' perceptions and experiences. Triangulation of the data in this study was made by inviting the input from a medical teacher from each of the two groups, i.e., active and passive healthy role models. MC and GRR also provided expert checking to determine whether the coding and representative quotations completely described the category and subcategory that were represented. All the identified subcategories, categories, and themes are presented in Table 1.

## Results

The results were classified into two themes: perceived facilitators and perceived barriers (Table 1). The intrinsic and extrinsic factors were identified as perceived facilitators and barriers that influenced medical teachers as a healthy role model in medical school to perform healthy behavior.

### Perceived facilitators

The intrinsic facilitators were motivation, conscious awareness, having physical limitation, knowledge, and economic reason. The impact of doing a particular job, feedback, time, and the environment were identified as the extrinsic facilitators. The intrinsic and extrinsic facilitators that support medical teachers as a healthy role model in medical school to perform healthy behavior were explained in more detail below with quotations.

**Table 1:** Classification of theme, categories, and subcategories

Subcategories	Categories	Theme
Motivation	Intrinsic facilitators	Perceived facilitators
Conscious awareness		
Having physical limitation		
Knowledge		
Economic reason		
The impact of doing a particular job	Extrinsic facilitators	
Feedback		
Time		
Environment		
The lack of self-motivation	Intrinsic barriers	Perceived barriers
Having physical limitation		
The burden of responsibilities for being medical teachers	Extrinsic barriers	
Environment		

#### Intrinsic facilitators

##### Motivation

Most participants stated that they had never desired to become a healthy role model in medical school. The healthy behavior that they perform in their daily lives is done by their motivation to become healthier.

"I have never thought to be a healthy role model in this school. I just want to be healthier...healthy for myself." (No. 2, female, Department of Ear, Nose, and Throat)

The interest in exploring something sometimes provokes them to conduct healthy behavior.

"In the Netherlands, I have three friends with bicycles. We like to explore new places by riding bicycles." (No. 10, male, Department of Microbiology)

##### Conscious awareness

All participants mentioned that due to working as medical teachers, they know how to maintain their health. However, this knowledge does not directly guarantee that they will automatically perform healthy behavior. They explained a conscious awareness is needed that health is an important life consideration, making healthy behavior one of their basic daily needs. This awareness is essential for medical teachers to perform healthy behavior consciously in their daily lives.

"I found Yoga is different from other physical activities. By doing Yoga, we have a benefit of all aspects of health-physical, mental, social, and spiritual. For example, in the mental health aspect, a Yogis is someone who can control emotions...Therefore, I always placed my Yoga schedule when I have work on the day I had a Yoga session." (No. 2, female, Department of Ear, Nose, and Throat)

"When I work out of town, I used sports facilities in the place where I stay. However, if they did not have facilities, I do only stretching in my room." (No. 5, female, Department of Health Nutrition)

##### Having physical limitations

Physical limitations caused by suffering a chronic disease and the increasing of age motivate the participants to perform healthy behavior routinely in their daily lives.

"If I do not have a physical exercise every day, my heart will not run normally as is needed because I have chronic heart disease." (No. 1, female, Department of Forensic Medicine and Medicolegal)

"In the afternoon, when I am going back home, I used to feel pain in my back. Now, when I have this routine of physical stretching exercises, I feel better." (No. 6, male, Department of Psychiatry)

##### Knowledge

The knowledge of health does not guarantee the medical teacher to perform healthy behavior in their daily life. The information about their current health status, the risk of suffering a particular chronic disease, and how to perform a healthy behavior effectively are needed to motivate the medical teachers to conduct healthy behavior.

"I knew that I had a heart problem when I participated in the research. It happened when I was a medical student. Knowing that disease, it was like finding a puzzle on why I usually feel faint since I was a child. Thus, I have to adopt some healthy behaviors to live with my chronic disease." (No. 1, female, Department of Forensic Medicine and Medicolegal)

"While working as a physician in the hospital, I realized that the elevator is one contaminated place. It is happening because it has only minimal ventilation. Therefore, I preferred to use emergency stairs, which unconsciously force me to behave healthily by using more calories when walking up the stairs." (No. 2, female, Department of Ear, Nose, and Throat)

##### Economic reason

The economic reason can also influence them in conducting healthy behavior in their daily life.

"In the Netherlands, in order to save money, I used to ride a bicycle to go everywhere in town." (No. 10, male, Department of Microbiology)

#### Extrinsic facilitators

##### The impact of doing a particular job

Some participants informed that they started to conduct healthy behaviors when they got a new assignment at their job.

"In 2014, when I was asked by the State Minister for Youth and Sports Affairs of Indonesia to accompany their athletes at the Football Association, I started to facilitate myself on doing a routine physical exercise because I was ashamed if I could not promote healthy behavior effectively to their athletes without any experience in doing those behaviors." (No. 5, female, Department of Health Nutrition)

##### Feedback

The feedback obtained from conducting healthy behavior has a significant influence on motivating medical teachers to keep doing such behaviors. This feedback could be the benefits of doing healthy behavior, other rewards, or a compliment from others.

"My family loves to participate in the marathon events around our home. We all got a medal. It motivated us to participated more in other events." (No. 3, female, Department of Anaesthesiology and Intensive Care)

##### Time

All of the participants considered their life as a heavy burden as the consequence for being medical teachers. Therefore, the availability of free time to conduct healthy behaviors becomes crucial.

"If other people asked me why I can have my running session every day, I have more time for myself. I remembered in the past several years when this city has only a small number of anesthesia doctors. I imagine how we have to manage all hospitals around this city with a small number of anesthesia doctors. The challenges come when our seniors must attend a seminar out of town. It exhausted us as the junior staff who must cover our senior responsibility as anesthesia doctors in the hospital they work." (No. 3, female, Department of Anaesthesiology and Intensive Care)

##### Environment

The environment contributes significantly. The influences included the presence of other people or communities who play a role as social influencers, social reminders, and role models; the availability of limited infrastructure such as no elevator services in the environment which unconsciously forces medical teacher to use stairs; the availability of policies in the environment that support people to conduct healthy behavior; the availability of media to promote healthy behavior in the environment; and the availability of facilities in the environment to support healthy behavior.

"From the last three years, this institution has changed its policy in serving snacks for meeting. Now, it serves more fruits as snacks. They also reduced the number of fatty foods for lunch." (No. 8, male, Department of Health Policy and Management)

"They put a sticker to inform how much calories we burn on each step we performed when taking stairs to go upstairs. It is successful to make many medical teachers start using stairs rather than an elevator." (No. 9, female, Department of Neurology)

### Perceived barriers

#### Intrinsic barriers

##### Lack of self-motivation

The heavy workload felt by medical teachers is strongly related to the decreasing in their motivation to engage in healthy lifestyles.

"Sometimes, I do not feel motivated to conduct healthy behavior because I am tired from finishing all my work at my job each day." (No. 9, female, Department of Neurology)

##### Having physical limitations

A limiting physical condition that medical teachers have sometimes becomes a barrier for them to conduct healthy behaviors.

"I had a cervical spine problem several years ago, and it forced me to stop my running activities for quite a while." (No. 3, female, Department of Anaesthesiology and Intensive Care)

#### Extrinsic barriers

##### The burden of responsibilities for being medical teachers

Most of the 24 hours that medical teachers have in each day are allocated to fulfill the responsibility of being staff in medical school. This time demand significantly reduces their time to take care of themselves, e.g., can decrease their motivation to perform physical exercise. Due to an increasing of assignments that institutions demand, many medical teachers are required in their job to devote all the time they have in a day and even sometimes at night. This heavy burden increased stress and finally can harm their health.

"We have a heavy burden of being a medical teacher. We have to teach, conduct research, make an excellent publication, and be involved in community activities, and we also have the routine meetings that almost filled out our entire schedule on the workday on campus. How can we provide time for joining a health activities program that is facilitated by this school?" (No. 10, male, Department of Microbiology)

"Having a full schedule in a day, it forces us to drive a car even though only moving from one building to a close building. This condition makes us to no longer be particularly devoted in our life to health." (No. 9, female, Department of Neurology)

##### Environment

Some situations happen in the environment which inhibit medical teachers from conducting healthy behavior. For example, there are the limitations to having access to sports facilities that are not available in the surrounding environment, they do not have support from the people close around them, the events of healthy behavior program are only conducted in at a specific time, the types of physical activity that are held in the environment did not accommodate personal interest, the lack of socialization of health facilities that are available in the environment, and the atmosphere in the surrounding environment is less conducive to conducting healthy behaviors.

"My brother and his family have overeating behavior. I always remind them, but it was not easy since they love to taste food in the new cafe." (No. 5, female, Department of Health Nutrition)

"They have a gymnastic session every Friday morning. However, I could not join because I have a teaching schedule at that time and also not interested in gymnastics." (No. 8, male, Department of Health Policy and Management)

From the analysis, we also found there were reasons for medical teachers to be healthy role models in medical schools that involved choosing one particular healthy behavior. Several reasons provoke medical teachers to choose one particular healthy behavior, such as an interest to adopt a particular healthy behavior, past healthy habits, and joining a community in one particular healthy behavior.

"I like vegetables and fruit so much. When someone invited me to have lunch or dinner, I preferred to choose a 'pecel,' 'or urap.' I do not need any effort to do that because it happens automatically. I did it because I love that food so much." (No. 1, female, Department of Forensic Medicine and Medicolegal)

"In the Department of Anaesthesiology and Intensive Care, we have many counselors that love running. Thus, we made a group to support each other." (No. 3, female, Department of Anaesthesiology and Intensive Care)

The differences among medical teachers as a healthy role model in the two groups: active and passive are identified in their levels of motivation and willingness to promote healthy behavior to others. The medical teachers in the active healthy role model group conduct healthy behavior as their daily habits. They make special arrangements for doing particular physical exercise, even when they have a busy schedule. Whereas, in the passive healthy role model group, the medical teachers only conduct healthy behavior when they have the free time. Furthermore, the active healthy role models conduct their physical exercise routinely. This regular activity then invites the attention of others to observe what they do. They also have a willingness to ask other people to join them in performing healthy behaviors when someone asked about their health behavior. This sense was captured in the interviews when they told us with enthusiasm about their experiences in inviting other people to perform healthy behavior together. This experience was not expressed by the participants from the passive healthy role models group because they were more focused on telling mostly about how their experiences were limited in conducting some kinds of healthy behaviors.

"I like physical activity. I love running so much." I remembered for the first time; it was only me, then finally I started to invite my husband then my children. Now, all of us always have running sessions together." (No. 3, female, Department of Anaesthesiology and Intensive Care, an active healthy role model)

"I used static bicycles at home for my physical exercise. It lasts for approximately 15 minutes in one session. However, I do not have this physical exercise regularly. It only happens when I have more free time." (No. 9, female, Department of Neurology, a passive healthy role model)

## Discussions

Our study identified the intrinsic and extrinsic facilitators that support medical teachers to conduct healthy behaviors. The motivation for doing healthy behavior cannot be separated from their belief in the benefits of doing the healthy behavior (self-efficacy).^14^ The benefits could be attributed to the physical and social aspects, and feedback information that are useful for making self-evaluation.^14^ In the physical aspect, some participants did not experience back pain when doing stretching exercises routinely. In the social aspect, some participants felt more confident when they see themselves appearing younger after taking yoga classes regularly. It makes them more confident to have social relations with others. In the self-evaluation aspect, some participants felt safer when they knew that regular physical activity could control symptoms of their disease. Medical teachers as healthy role models in medical school need a sense of self-empowerment or self-efficacy. Someone who has high self-efficacy tends to have the belief that everything they do depends on themselves.^15^^-^^17^ Therefore, with this self-efficacy, they can handle any barriers that may occur in performing healthy behaviors.

Some of our participants have a chronic disease. It makes them aware that they are vulnerable to getting an illness. Accordingly, they are motivated to conduct healthy behaviors. In the Health Belief Model (HBM),^18^ people who realize that they are vulnerable to a particular disease that can harm their lives, and have a belief that doing healthy behavior could prevent them from the risk of suffering that disease, will adopt the healthy behavior they believe will help. The benefits which our participants get from doing one healthy behavior make them have a higher self-efficacy in doing healthy behavior. However, some chronic diseases could lead to a physical limitation condition; thus, it will cause a barrier to healthy behavior. The presence of physical limitations caused by chronic disease also causes a decrease in a medical teacher's self-efficacy, especially when the person lacks the self-motivation to conduct healthy behavior.

The knowledge of health gained as a medical teacher only acts as a prerequisite for health behavior change, because it increases the self-awareness that they are vulnerable to the risk of illness or increases their attention to observe their healthy colleagues.^19^ However, this knowledge is still needed in creating a healthy behavior change.^14^ Among respondents, the people who lack the knowledge of their vulnerability to a particular disease tended to experience some difficulty to change their unhealthy behavior.

Our findings show the economic reason was one of the factors that support medical teachers to conduct healthy behaviors. This finding is different from other studies outside the context of medical schools. Those studies found that low-income influences the more significant practice of unhealthy living behaviors, such as low or no physical activity,^20^^, ^^21^ and unhealthy eating habits.^22^^-^^24^ Humphreys and Ruseski^20^ aimed to explore how a person's economic factors influence their active participation in physical activity. They found that the higher the amount of income that someone has would increase their tendency to actively participate in physical activity. Kari and colleagues^21^ conducted a study to determine the relationship between the amount of a person's income and physical activity conducted every day, the physical activity done during the free time, and the number of footsteps in one day measured by a pedometer by 753 adults in Finland. They found a strong relationship between the amount of income and physical activity, but only in the female participants of the study and not for men. Appelhans and colleagues^22^ conducted a cross-sectional study among visitors who shop at a supermarket and found that visitors with low income tend to buy more fattening foods that are less nutritional. French and colleagues^23^ studied the relationship between the amount of income in a household and the quality of food purchased. Their results showed that households with low incomes tend to buy unhealthy food. Pechey and Monsivais^24^ assessed households' economic status with food choices purchased from supermarkets. They found that households with higher economic status tend to buy healthy food. However, our participants' characteristics in this study that are different from the other studies might result in some differences.

In this study, we also found extrinsic facilitators have a significant impact on supporting medical teachers to conduct healthy behavior. The environment, as one of the extrinsic facilitators, is explained by Bandura's social cognitive theory.^8^ According to Bandura, human behavior is influenced by its interactions with personal factors and the environment, known as reciprocal determinism. The presence of medical teachers as a healthy role model in the medical school environment serve as a social influencer, social reminder, and role model to students and staff. Several studies found that parents, friends, and teachers can also play a role as social influencers,^25^ social reminders^26^ and role models.^26^^-^^30^ We used these studies since no publication was found regarding healthy role models in the medical school context. Cheng, Mendoca, and de Farias Junior^25^ conducted a cross-sectional study of adolescents aged 14-19 years to assess the relationship between physical activity frequency and social support originating from parents and friends. They found that social support increased self-efficacy in children. Tibs and colleagues^26^ interviewed African-American parents to evaluate the parents' frequency in modeling healthy eating behaviors for their children. They found that African-American parents did not only play a role model by modeling healthy eating foods to their children. Additionally, they also shared lunchtime together with their children when they want to model some new healthy food. Angoorani and colleagues^27^ surveyed to evaluate the relationship between parental weight status and physical activity with television viewing habits in their children. They found that children who have obese parents tend to engage in sedentary activities. Brunet and colleagues^28^ conducted a longitudinal study on 190 adolescents and found an increase in adolescents' physical activity when their parents were actively involved in their physical activities. Cheney, Oman, and Vesely^29^ conducted a longitudinal study on 467 pairs of parents and teenagers aged 12-17 years and described that other family members in a household who are similar in age as the children could reduce the frequency of smoking behavior in adolescents. Layzer, Rosapep, and Barr^30^ conducted interviews to evaluate the effectiveness of safe sex learning in adolescents using adolescent models as peer educators who were older than the subjects. They found that more participants conducted this healthy behavior because they were motivated to practice what they learned from their peers. The presence of parents and peers who modeled healthy behavior has been proved to increase the self-efficacy of observers to conduct healthy behaviors by mediating as a social influencer, social reminder, and healthy role model.^15^^, ^^17^

To be an active healthy role model, a medical teacher must have experiences in conducting healthy behavior actively. Active healthy role models should also appropriately consult about challenges when doing healthy behavior. Their shared experiences of doing healthy behavior, especially the strategies to maintain their self-efficacy for healthy behavior, are important to support their being active healthy role models in medical school. Whittaker and colleagues^31^ found that the effectiveness of smoking cessation programs in adolescents occurred because the program was facilitated by role models who have experience in smoking cessation. Parent and Fortin^32^ also found that the effectiveness of compliance of patients undergoing coronary artery bypass graft to attend a rehabilitation program occurred only in the group accompanied by a role model with the same treatment history who had succeeded through the rehabilitation program to have regular daily activities. Our study showed that the active healthy role models have the experience that some of their colleagues used them as consultants when they have a problem doing the same healthy behavior as healthy role models do.

Voorhees and colleagues also identified the role of the environment in facilitating healthy behavior.^33^ They found that public facilities that could be used both for recreation and having physical activities would increase the intention of people to use that facility. Also, the regulation that obligates people to conduct healthy behavior has proved to increase people's intention to conduct healthy behaviors.^34^ Our study found that the presence of new regulations on changing to healthier snacks at faculty meetings unconsciously developed into a new healthy habit of the medical teachers.

Furthermore, the role of media to promote healthy behaviors available in the environment effectively increases people's intention to perform healthy behavior. The study of Pappas-DeLuca and colleagues^35^ and Whittaker and colleagues^31^ showed that the media effectively promotes healthy behavior to their participants. Pappas-DeLuca and colleagues^35^ surveyed 555 adolescent participants in Botswana to evaluate the effectiveness of radio broadcast programs to promote HIV screening testing. They found that the promotional broadcast program increased the enthusiasm of participants to conduct HIV screening examinations. Whittaker and colleagues^31^ conducted an experimental study with a randomized controlled trial to evaluate the effectiveness of smoking cessation promotion using mobile media in teenagers and young adults aged 16-25. They found that media is effective in promoting smoking cessation behavior in teenagers and young adults. In our results, we identified that wall stickers which informed the number of calories used per step when climbing stairs increased the tendency of medical teachers to choose stairs over using the elevator.

The main barrier for medical teachers to conduct healthy behavior in this study is their workload, which diminished the leisure time for medical teachers to take care of themselves. The decrease in leisure time will lower their motivation to conduct healthy behavior because the remaining time is preferably used to rest. The lack of time to conduct healthy behavior is a barrier to prevent people from having healthy behavior, as explained by the theory of planned behavior.^36^ Even though the lack of time only has a short-term effect by causing delays in practicing healthy behavior, when this issue is maintained for a long time, it will decrease motivation to perform healthy behavior. A study of Wright and Caresse^37^ and mentioned by Crues, Cruess, and Steinert^10^ explained that the workload of medical teachers is a primary factor that inhibits the effectiveness of role modeling in medical schools. Our participants explained the increase of academic qualifications for medical teachers directly increases the number of assignments given to them by the institution. Therefore, oftentimes medical teachers ultimately feel stuck in their life only for ‘all work and no play’, which will tend to increase physical fatigue, lack of leisure time, stress, and decrease their motivation to conduct healthy behavior.

In this study, one of the three reasons for a medical teacher to choose a particular health behavior is past healthy habits. Some of our participants shared their history to explain the reasons to choose their present healthy behavior. Verplanken and Aarts^38^ and Ajzen^39^ explained that past behavior directly influences future behavior through repetition. The patterned behavior must occur continuously in a stable condition. When the same situation occurs, the production of behavior will automatically happen, and this process occurs unconsciously. By better understanding this process, we could explain the relationship between the habitual factor and the healthy behavior adopted by our participants.

The difference between the characteristics of the two groups of healthy role models (active and passive) found in this study also supports the interpretation of our developed questionnaires in the previous survey.^12^ The difference between active and passive healthy role models emphasizes healthy modeling characteristics. A passive healthy role model tends to not have the willingness to promote health behavior actively to others. Furthermore, the inconsistent time to conduct healthy behavior in the passive healthy role model group did not attract others’ attention to observe their healthy behavior, which is essential to initiate the modeling process.^8^^, ^^14^ As a consequence, healthy modeling could not occur adequately in the passive healthy role model group. This pattern has serious implications for future medical professionals in any ‘walk of life’.

### Limitations

The limitations in this study included the limited number of subjects which poses a challenge to generalize these results. Without more participants, it was difficult to explain the comprehensive factors that support and inhibit medical teachers in medical school to conduct healthy behaviors. Also, other factors were not explained in this study, e.g., personality traits that might influence medical teachers to conduct healthy behavior. Several studies explained that some personality traits (conscientiousness and extraversion) have a relationship with healthy behavior. For examples, Siegler, Feaganes, and Plaeffli^40^ found that the conscientiousness trait affected the patient's obedience to mammography treatments. Additionally, Rhodes and Plaeffli^41^^, ^^42^ found that individuals with the extraversion trait had a high intensity of physical activity. Hagger-Johnson and Shickle^43^ found that a person with conscientiousness trait tends to have more frequent physical activity, consume less alcoholic drinks, the low tendency of using illegal drugs, and rarely engage in dangerous sexual behavior. Hampson and collegeaus^42^ identified that people with extraversion and conscientiousness traits are easier to motivate in smoking cessation. Considering the results of these previous studies about the influence of personal traits, we suggest that future research about healthy role models in medical schools should include these aspects.

## Conclusions

Our participants acknowledged factors that serve as facilitators and barriers for a medical teacher as a healthy role model in medical schools to conduct healthy behavior. Understanding these factors could be considered in the planning and designing of the appropriate interventions, mainly by focusing on removing barriers and strengthening facilitators to increase the effectiveness of the characteristics of medical teachers as healthy role models. By doing this, medical schools can continue to contribute to increasing the health of a nation by producing healthy physicians who serve as healthy role models in their community.

### Acknowledgments

The authors wish to thank all participating medical teachers from the Faculty of Medicine, Public Health, and Nursing, Universitas Gadjah Mada, Indonesia, especially from Department of Anesthesiology and Intensive Therapy, Department of Ear, Nose, and Throat (ENT), Department of Forensic Medicine and Medicolegal, Department of Pharmacology and Therapy, Department of Health Nutrition, Department of Neurology, Department of Microbiology, Department of Histology; Department of Health Policy and Management, and Department of Psychiatry for their time and opinions to provide us valuable insights on this topic. We also thank drg. Natalya Wijaya, who helped as a coder. We also thank Lisbet, who helps as a transcriber.

### Conflict of Interest

The authors report no conflict of interest. The authors alone are responsible for the content and writing of the article.

## Supplementary material

Supplementary file 1 Appendix 1. Interview Guide
