# Supplementary material for: Factors influencing healthy role models in medical school to conduct healthy behavior: a qualitative study
Source: Int J Med Educ. 2021 Jan 22;12:1–11. doi: 10.5116/ijme.5ff9.9a88 (PMC7883803; doi:10.5116/ijme.5ff9.9a88)
Supplement: Supplementary file 1 — Appendix 1. Interview Guide [file ijme-12-1-S1.pdf]

Appendix 1.

Interview Guide

Semi-structured Questionnaire

1. Do you have the desire to be a healthy role model in medical school?
2. What kind of healthy behaviors do you do in your daily life?
3. Did you share your gaining experiences in performing healthy behaviors?
4. Does this institution provide a pleasant environment for you to conduct healthy behaviors effectively?
5. What factors that this institution has in order to support you in conducting healthy behavior?
6. What inhibition factors that challenge you to conduct healthy behavior?
7. Are there any recommendations to maximize the effectiveness of this institution in supporting their medical teachers for conducting healthy behavior?

All of the above questions are then explored again using the words 'What,' 'Why,' and 'How.'
